# Left ventricular fibrosis and hypertrophy are associated with mortality in heart failure with preserved ejection fraction

**DOI:** 10.1038/s41598-020-79729-6

**Published:** 2021-01-12

**Authors:** Pankaj Garg, Hosamadin Assadi, Rachel Jones, Wei Bin Chan, Peter Metherall, Richard Thomas, Rob van der Geest, Andrew J. Swift, Abdallah Al-Mohammad

**Affiliations:** 1 Department of Infection, Immunity and Cardiovascular Disease, University of Sheffield, Sheffield, S10 2RX UK; 2 Sheffield Teaching Hospital NHS Foundation Trust, Sheffield, UK; 3 Leiden University Medical Centre, Leiden, The Netherlands; 4 Norwich Medical School, University of East Anglia, Norwich, UK

**Keywords:** Cardiology, Heart failure, Outcomes research

## Abstract

Cardiac magnetic resonance (CMR) is emerging as an important tool in the assessment of heart failure with preserved ejection fraction (HFpEF). This study sought to investigate the prognostic value of multiparametric CMR, including left and right heart volumetric assessment, native T1-mapping and LGE in HFpEF. In this retrospective study, we identified patients with HFpEF who have undergone CMR. CMR protocol included: cines, native T1-mapping and late gadolinium enhancement (LGE). The mean follow-up period was 3.2 ± 2.4 years. We identified 86 patients with HFpEF who had CMR. Of the 86 patients (85% hypertensive; 61% males; 14% cardiac amyloidosis), 27 (31%) patients died during the follow up period. From all the CMR metrics, LV mass (area under curve [AUC] 0.66, SE 0.07, 95% CI 0.54–0.76, p = 0.02), LGE fibrosis (AUC 0.59, SE 0.15, 95% CI 0.41–0.75, p = 0.03) and native T1-values (AUC 0.76, SE 0.09, 95% CI 0.58–0.88, p < 0.01) were the strongest predictors of all-cause mortality. The optimum thresholds for these were: LV mass > 133.24 g (hazard ratio [HR] 1.58, 95% CI 1.1–2.2, p < 0.01); LGE-fibrosis > 34.86% (HR 1.77, 95% CI 1.1–2.8, p = 0.01) and native T1 > 1056.42 ms (HR 2.36, 95% CI 0.9–6.4, p = 0.07). In multivariate cox regression, CMR score model comprising these three variables independently predicted mortality in HFpEF when compared to NTproBNP (HR 4 vs HR 1.65). In non-amyloid HFpEF cases, only native T1 > 1056.42 ms demonstrated higher mortality (AUC 0.833, p < 0.01). In patients with HFpEF, multiparametric CMR aids prognostication. Our results show that left ventricular fibrosis and hypertrophy quantified by CMR are associated with all-cause mortality in patients with HFpEF.

## Introduction

Heart failure with preserved ejection fraction (HFpEF) is a very common clinical syndrome with high morbidity and mortality. HFpEF accounts for approximately half of all clinical heart failure (HF) presentations. HFpEF is an emerging epidemic which is increasing over time, especially with the rise in the number of older people who also have a large number of comorbidities^1^. The most common cause of death in HFpEF are cardiovascular causes, making up to 60% of deaths in epidemiological studies and > 70% in clinical trials^2^.

Data from several studies suggested that focal or diffuse fibrosis have been involved with the pathophysiology of HFpEF. This occurs through stimulating adverse ventricular remodelling, increasing myocardial stiffness and thus contributing to diastolic dysfunction^3^. In addition, increased left ventricular (LV) mass can solely predict adverse outcomes^4,5^. Neurohumoral activation, mechanical overload, increased release of cytokines in response to arterial hypertension, CKD, DM and other comorbidities contribute to LV hypertrophy (LVH)^6^.

Cardiac magnetic resonance (CMR) imaging is the gold standard for quantification of left ventricular ejection fraction (LVEF), confirming left atrial enlargement and LVH^7^. Another significant aspect of CMR is the ability to sub-phenotype HFpEF patients through multiparametric tissue characterisation and first-pass perfusion, besides its volumetric assessment capabilities^8^. Sub-phenotyping has been instrumental in our understanding of HFpEF and the development of promising therapies^9^. Late gadolinium enhancement (LGE) and native T1-mapping are valuable CMR tools for detection of myocardial fibrosis, infiltration and scar^10^. While LGE can only detect focal myocardial fibrosis, T1 mapping is able to identify diffuse fibrosis. However, the role of native T1-mapping in HFpEF remains unclear.

In this study, we hypothesise that multiparametric CMR consisting of volumetric assessment, fibrosis/scar assessment by LGE and diffuse fibrosis assessment by native T1-value will have prognostic value in patients with HFpEF. Accordingly, the main objective of this study was to investigate the prognostic value of multiparametric CMR, including left and right heart volumetric assessment, native T1-mapping and LGE in HFpEF.

## Methods

### Study design

This is a single-centre retrospective study. It includes patients who had a confirmed clinical and echocardiographic diagnosis of HFpEF as per the National Institute of Clinical Excellence (NICE) in the United Kingdom and the European Society of Cardiology guidelines^11,12^. To be included in the study, the patient must be > 18 years of age, has confirmed diagnosis of HFpEF and had undergone a CMR. As part of the routine clinical work-up in out-patient heart failure clinics, patients received NTproBNP test. NTproBNP tests preceded the CMR scans which were also requested on clinical grounds in the clinic. Exclusion criteria: previous permanent pacemaker, defibrillator implantation or any other contraindication to CMR imaging including claustrophobia and end-stage renal impairment (eGFR < 30).

### Ethics approval

This study was approved by the National Research Ethics Service (REC reference: 17/YH/0142), written consent was waived for this study. The study complied with the Declaration of Helsinki.

### CMR protocol

**Figure 1:**
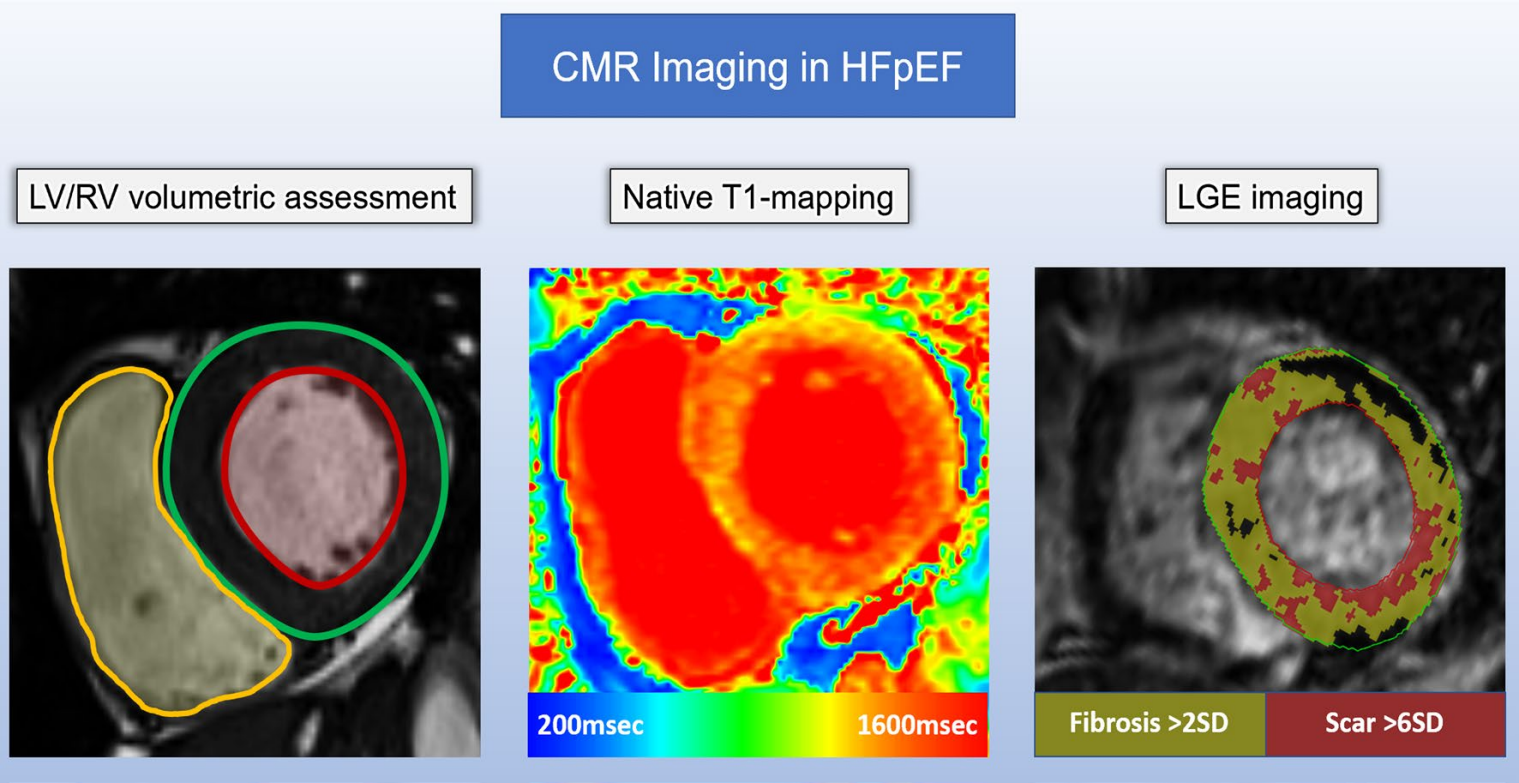
Central illustration to demonstrate the value of multiparametric cardiovascular magnetic resonance imaging for informing prognosis. In this case example, there is evidence of concentric LV hypertrophy on cine imaging, the native T1 is significantly raised throughout, and there are focal areas of scar and fibrosis on LGE imaging.

All CMR imaging was performed on a 1.5 T system (Magnetom Avanto, Siemens Healthcare, Erlangen, Germany) using an eighteen-channel cardiac phased-array receiver. The protocol included cines, native T1-mapping and late gadolinium enhancement imaging (Fig. 1)^13^.

### Cines

Steady-state free precession (SSFP) cine images were obtained during repeated breath-holding in two long-axis views (two chambers and four chambers) and, in a stack of short-axis views covering the LV for quantification of cardiac chamber volumes and function^14–17^. Short-axis LV stack was acquired using a cardiac gated multislice balanced SSFP sequence (30 frames per cardiac cycle, slice thickness 6 mm, FOV matrix 360 × 360, GRAPPA acceleration factor 2, TR/TE 38.92/1.13 ms). Patients were scanned in the supine position with a surface coil and retrospective electrocardiogram gating.

### Native T1-mapping

Mid-ventricular native T1 maps were acquired using the following sequence: a Modified Look-Locker Inversion Recovery (MOLLI)^18^. The acquisition parameters were: pixel bandwidth 1085 Hz/pixel; echo time = 1.13 ms; flip angle = 35°; TI = 180 ms; matrix = 256 × 144; slice thickness = 8 mm^19^. Inline motion correction and a non-linear least-square curve fitting were performed using the vendor-provided motion correction algorithm, (MyoMaps) with the set of images acquired at different inversion times to generate a pixel-wise coloured T1 map.

Quality control was performed during scanning by reviewing the "goodness of fit" map and source images to allow an immediate repetition of suboptimal measurements to minimise the respiratory motion and off-resonance effects.

### Late gadolinium enhancement

LGE imaging performed 10 min post-contrast (Gadovist, dosed at 0.1 mls per kg body weight) using 2 breath-held methods in 3 stacks of contiguous slices encompassing the whole left ventricle in short-axis, 2-chamber, and 4-chamber orientations.

### Image analysis

All images were evaluated offline using in-house developed research software (MASS; Version 2019-EXP, Leiden University Medical Center, Leiden, The Netherlands).

Right and left endocardial and epicardial surfaces were manually traced from the stack of short-axis cine images to obtain left ventricular (LV) end-diastolic volume (LVEDV), LV end-systolic volume (LVESV), right ventricular (RV) end-diastolic volume (RVEDV) and RV end-systolic volume (RVESV). From end-diastolic and end-systolic volumes, LV stroke volume (LV SV), LV ejection fraction (LV EF), RV stroke volume (RV SV) and RV ejection fraction (RV EF) were calculated. For the calculation of ventricular mass, the interventricular septum was considered as part of the LV. RV mass (RV mass) and LV mass (LV mass) were derived in end-diastole^15,20^.

Region of interest was drawn in the mid interventricular septum avoiding any partial voluming area on native T1 maps. The mean value of ROI was recorded.

For scar and fibrosis assessment on late gadolinium enhancement, semi-automated methods of standard deviation (SD) of the signal intensity of the remote normal myocardium were applied after epi and endocardial segmentation of the left ventricle. For myocardial scar, > 6SD of the normal myocardial signal intensity was used, and for myocardial fibrosis assessment, > 2SD of the normal myocardial signal intensity was used.

### Statistics

Statistical analysis was performed in SPSS version 22 (IBM, Chicago, USA) and confirmed in MedCalc (MedCalc Software, Ostend, Belgium version 19.1.5). Graphing was undertaken in Origin Lab Pro (Origin Lab Corp., Northampton, MA). Data were treated as normally distributed. Continuous variables were presented as mean ± standard deviation. Categorical data were reported as frequencies and percentages.

A two-sample independent t-test was used to compare normally distributed continuous variables. The Chi-squared test was used for categorical data.

Prognostic performance of each clinical characteristics and CMR metric was done using the receiver operator characteristic (ROC) statistics to compute the area under the curve (AUC). Cut-off values for variables which demonstrated association were done using Youden's J statistics where applicable. ROC analysis was used to obtain the sensitivity and specificity for different values. A CMR score model was developed to integrate all variables which demonstrated an association to mortality. For each variable, if the value was greater than the cut-off, a score of 1 was registered. The final score was some of all scores for individual variables. Kaplan Meier analysis and Cox proportional hazard model was used for univariate and multivariate analysis of prognosis. Stepwise regression method was used to investigate the incremental clinical role of proposed model. Propensity matched Kaplan Meier curves were generated adjusting for any clinical parameters associated with outcome. Unless otherwise stated, all statistical tests were two-tailed, and a p-value of < 0.05 was deemed significant.

### Consent for publication

All authors have read and approved the final version of this manuscript.

## Results

The demographic data and CMR characteristics of patients are shown in Tables 1 and 2. During a mean follow up period of 3.2 + 2.4 years, 27 of 86 patients (31%) reached the endpoint of all-cause mortality. There were no statistical differences between those patients who were alive and those who died in terms of sex or comorbid history. Nearly one-third of patients had a history of atrial fibrillation (24 of 59 alive vs 6 of 27 dead). There was a high prevalence of systemic hypertensive disease (84%), followed by coronary artery disease (37%). Nearly half of the patients were smokers, and a substantial minority were either diabetic or had a previous myocardial infarction (12%). 4 out of 27 patients who died had COPD, and 10 (17%) didn't reach the endpoint.

**Table 1:** Study demographics.

	Alive n = 59	Dead n = 27	p-value
Age (years)	77 ± 9	78 ± 10	0.50
Male sex	36 (61)	14 (52)	0.43
BMI	31 ± 8	27 ± 5	0.03
Atrial fibrillation	24 (41)	6 (22)	0.10
Hypertension	50 (85)	22 (81)	0.71
Coronary artery disease	19 (32)	13 (48)	0.16
Myocardial infarction	8 (14)	3 (11)	0.76
Diabetes mellitus	12 (20)	3 (11)	0.33
COPD	10 (17)	4 (15)	0.81
Smoker	27 (46)	11 (41)	0.67
Amyloid	4 (7)	8 (30)	< 0.01
Haemoglobin (g/L)	127.6 ± 15.9	124.3 ± 12.6	0.36
Blood urea (mmol/L)	9.2 ± 4.4	9.8 ± 5.8	0.63
Serum creatinine (µmol/L)	101.1 ± 32.5	116.2 ± 41.6	0.08
eGFR (mL/min/1.73 m^2^)	55.8 ± 16.6	52 ± 18.7	0.35
NTproBNP (pg/mL)	1775.7 ± 1351.6	3868.9 ± 3600.2	< 0.01

**Table 2:** Left and right heart functional CMR assessment.

	Alive n = 59	Dead n = 27	p value
**Left heart assessment**			
LV end-diastolic volume (mL)	147.5 ± 41	149 ± 34.2	0.87
LV end-systolic volume (mL)	61.5 ± 28.1	62.6 ± 20.8	0.86
LV stroke volume (mL)	85.9 ± 27.2	86.4 ± 17.6	0.94
LV ejection fraction (%)	59 ± 12.4	58.8 ± 7.2	0.93
LV mass (g)	126.3 ± 39.4	148 ± 42.3	0.03
Scar core (%)	8.3 ± 7.1	10.9 ± 11.5	0.23
Fibrosis (%)	30.7 ± 10	36.9 ± 10.9	0.02
Native T1-values (ms)	1064.1 ± 52	1107.2 ± 39.5	0.09
**Right heart assessment**			
RV end-diastolic volume (mL)	143.1 ± 59.4	149.9 ± 48.2	0.62
RV end-systolic volume (mL)	68.2 ± 42	68.5 ± 25.4	0.97
RV stroke volume (mL)	78.4 ± 27.7	81.4 ± 27.6	0.66
RV ejection fraction (%)	55.3 ± 13.8	54.4 ± 8	0.77
RV Mass (g)	39.7 ± 21.6	40.5 ± 12	0.86

The clinical characteristics which were significantly different in HFpEF patients who died during the study were body mass index (BMI), the finding of cardiac amyloidosis on CMR and the level of N-terminal pro-Brain-type Natriuretic peptide (NTproBNP). Thus, 8 out of 27 (15%) patients who died had amyloidosis, which was statistically different as only 4 of 59 (7%) of the alive group had amyloidosis. NTproBNP was significantly higher in patients who died during the FU period (p < 0.01).

As shown in Table 2, no significant correlation was found regarding left and right heart volumetric assessment and all-cause mortality except for LV mass. Patients who died had greater LV mass (148 ± 42.3 vs 126.3 ± 39.4, p = 0.03). In addition, LV fibrosis by LGE was significantly greater in the patients who died during follow up compared to those who remained alive (36.9 ± 10.9 vs 30.7 ± 10, p = 0.02). Also, native T1 demonstrated a trend to be higher in HFpEF patients who died (1107.2 ± 39.5 vs 1064.1 ± 52, p = 0.09).

**Figure 2:**
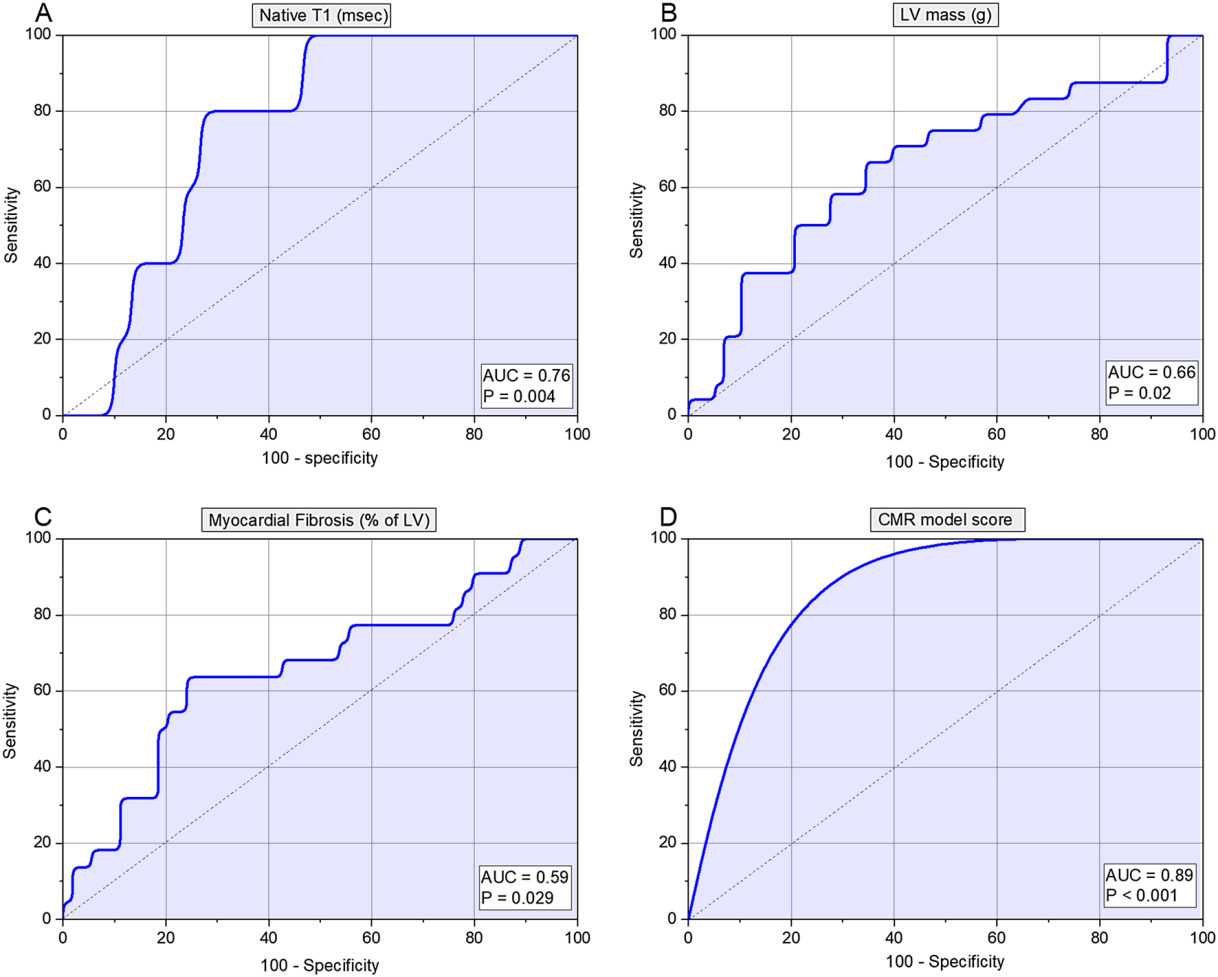
Receiver operator characteristics (ROC) results for CMR functional parameters.

In receiver operator characteristic (ROC) analysis, the three CMR metrics, which demonstrated significant area under the curve (AUC), were LV mass (AUC 0.66, p = 0.02), fibrosis (AUC 0.59, p = 0.03) and native T1-values (AUC 0.76, p < 0.01) (Table 3, Fig. 2). The optimum thresholds for LV mass was > 133.24 g (HR 1.58, 95% CI 1.1–2.2, p < 0.01), for focal fibrosis on LGE volume the percentage of > 34.86% (HR = 1.77, 95% CI 1.1–2.8, p = 0.01) and for native T1-values a time of > 1056.42 ms (HR 2.36, 95% CI 0.9–6.4, p = 0.07). Moreover, we developed a CMR score model to integrate all three variables associated with mortality. For each variable, if the value was greater than the cut-off, a score of 1 was registered (HR 4, 95% CI 1.2–13.9, p < 0.001) (Table 4).

**Table 3:** Univariate regression and receiver operator characteristics (ROC) results for all study parameters.

	HR	95% CI of HR	AUC	95% CI of AUC	p
**Clinical characteristics**					
Age (years)	1.21	0.7846–1.8618	0.566	0.429–0.702	0.34
Gender	0.61	0.2828–1.3338	0.55	0.431–0.661	0.44
BMI (kg/m^2^)	0.45	0.2457–0.8179	0.66	0.535–0.781	0.01
Atrial fibrillation	0.45	0.1687–1.1914	0.59	0.490–0.694	0.07
Hypertension	1.00	0.3465–2.9027	0.52	0.428–0.604	0.71
Coronary artery disease	1.95	0.9006–4.2043	0.58	0.466–0.693	0.17
Myocardial infarction	0.76	0.2271–2.5276	0.51	0.437–0.587	0.75
Diabetes mellitus	0.54	0.1614–1.8250	0.54	0.463–0.625	0.29
COPD	1.00	0.3401–2.9363	0.51	0.427–0.594	0.8
Smoker	0.91	0.4131–2.0253	0.53	0.411–0.639	0.66
Haemoglobin (g/L)	0.78	0.5296–1.1604	0.57	0.440–0.692	0.31
Blood urea (mmol/L)	1.18	0.8060–1.7272	0.51	0.372–0.643	0.91
Serum creatinine (µmol/L)	1.58	1.0805–2.3144	0.61	0.476–0.741	0.11
eGFR (mL/min/1.73 m^2^)	0.86	0.5591–1.3324	0.58	0.442–0.711	0.27
NTproBNP (pg/mL)	1.66	1.2921–2.1227	0.69	0.56–0.83	0.004
**Left heart assessment**					
LV end-diastolic volume (mL)	1.20	0.7840–1.8257	0.54	0.430–0.654	0.54
LV end-systolic volume (mL)	1.22	0.8049–1.8445	0.54	0.429–0.653	0.54
LV stroke volume (mL)	1.08	0.7090–1.6486	0.50	0.388–0.613	0.99
LV ejection fraction (%)	0.91	0.5936–1.3890	0.55	0.439–0.663	0.42
LV mass (g)	1.58	1.1238–2.2233	0.66	0.548–0.762	0.02
Scar core (%)	1.45	0.9483–2.2026	0.69	0.508–0.832	0.77
Fibrosis (%)	1.45	0.9483–2.2026	0.59	0.415–0.756	0.03
Native T1-values (msec)	2.36	0.8767–6.3768	0.76	0.586–0.888	< 0.01
**Right heart assessment**					
RV end-diastolic volume (mL)	1.12	0.7468–1.6649	0.80	0.631–0.916	0.37
RV end-systolic volume (mL)	1.09	0.7534–1.5685	0.82	0.654–0.929	0.42
RV stroke volume (mL)	0.98	0.6225–1.5288	0.69	0.515–0.837	0.75
RV ejection fraction (%)	0.76	0.5085–1.1441	0.54	0.424–0.649	0.55
RV Mass (g)	1.05	0.7531–1.4680	0.59	0.479–0.701	0.20

**Table 4:** Clinically relevant cut-offs for CMR variables including the CMR score model.

	Cut-offs	HR	95% CI		p-value
LV mass (g)	> 133.24	1.58	1.1	2.2	< 0.01
Fibrosis (%)	> 34.86	1.77	1.1	2.8	0.01
Native T1-values (msec)	> 1056.42	2.36	0.9	6.4	0.07
CMR score model	> 1	4	1.2	13.9	< 0.001

In HFpEF patients without cardiac amyloidosis, only BMI and native T1 demonstrated association to mortality (Table 5). NTproBNP still had a strong trend to be higher in patients who died during the FU period.

**Table 5:** Univariate regression and receiver operator characteristics (ROC) results for the study cohort excluding amyloid cases.

Covariate	HR	95% CI of HR	AUC	95% CI of AUC	p
**Clinical characteristics**					
Age (years)	1.35	0.7957–2.3037	0.581	0.426–0.737	0.31
Gender	0.66	0.2606–1.6952	0.538	0.418–0.654	0.57
BMI (kg/m^2^)	0.40	0.1866–0.8493	0.662	0.539–0.771	0.03
Atrial fibrillation	0.53	0.1745–1.6233	0.568	0.448–0.683	0.27
Hypertension	0.95	0.2749–3.2910	0.533	0.413–0.650	0.54
Coronary artery disease	2.52	0.9949–6.4070	0.618	0.497–0.728	0.08
Myocardial infarction	1.22	0.3516–4.2249	0.524	0.405–0.642	0.61
Diabetes mellitus	0.49	0.1120–2.1794	0.554	0.433–0.670	0.26
COPD	1.16	0.3282–4.0880	0.503	0.384–0.621	0.95
Smoker	0.85	0.3294–2.2152	0.526	0.406–0.643	0.7
Haemoglobin (g/L)	0.75	0.4685–1.1959	0.599	0.478–0.713	0.16
Blood urea (mmol/L)	1.01	0.6263–1.6288	0.506	0.386–0.625	0.94
Serum creatinine (µmol/L)	1.35	0.8511–2.1292	0.573	0.451–0.688	0.37
eGFR (mL/min/1.73 m^2^)	0.75	0.4543–1.2269	0.573	0.451–0.688	0.35
NTproBNP (pg/mL)	1.72	1.2886–2.2894	0.668	0.543–0.779	0.06
**Left heart assessment**					
LV end-diastolic volume (mL)	1.23	0.7460–2.0413	0.541	0.420–0.659	0.62
LV end-systolic volume (mL)	1.20	0.7316–1.9687	0.54	0.419–0.658	0.61
LV stroke volume (mL)	1.15	0.6940–1.8971	0.509	0.389–0.629	0.9
LV ejection fraction (%)	0.96	0.5764–1.6117	0.548	0.427–0.666	0.49
LV mass (g)	1.44	0.9549–2.1860	0.598	0.476–0.712	0.23
Scar core (%)	0.88	0.4845–1.5961	0.575	0.446–0.697	0.41
Fibrosis (%)	1.33	0.7816–2.2540	0.569	0.441–0.692	0.44
Native T1-values (ms)	2.84	1.0585–7.6449	0.833	0.656–0.942	< 0.01
**Right heart assessment**					
RV end-diastolic volume (mL)	1.23	0.7629–1.9865	0.573	0.451–0.689	0.4
RV end-systolic volume (mL)	1.18	0.7729–1.7983	0.559	0.437–0.676	0.47
RV stroke volume (mL)	1.04	0.6149–1.7567	0.544	0.423–0.662	0.59
RV ejection fraction (%)	0.75	0.4639–1.2231	0.558	0.436–0.675	0.4
RV Mass (g)	1.02	0.6748–1.5478	0.551	0.430–0.669	0.55

**Figure 3:**
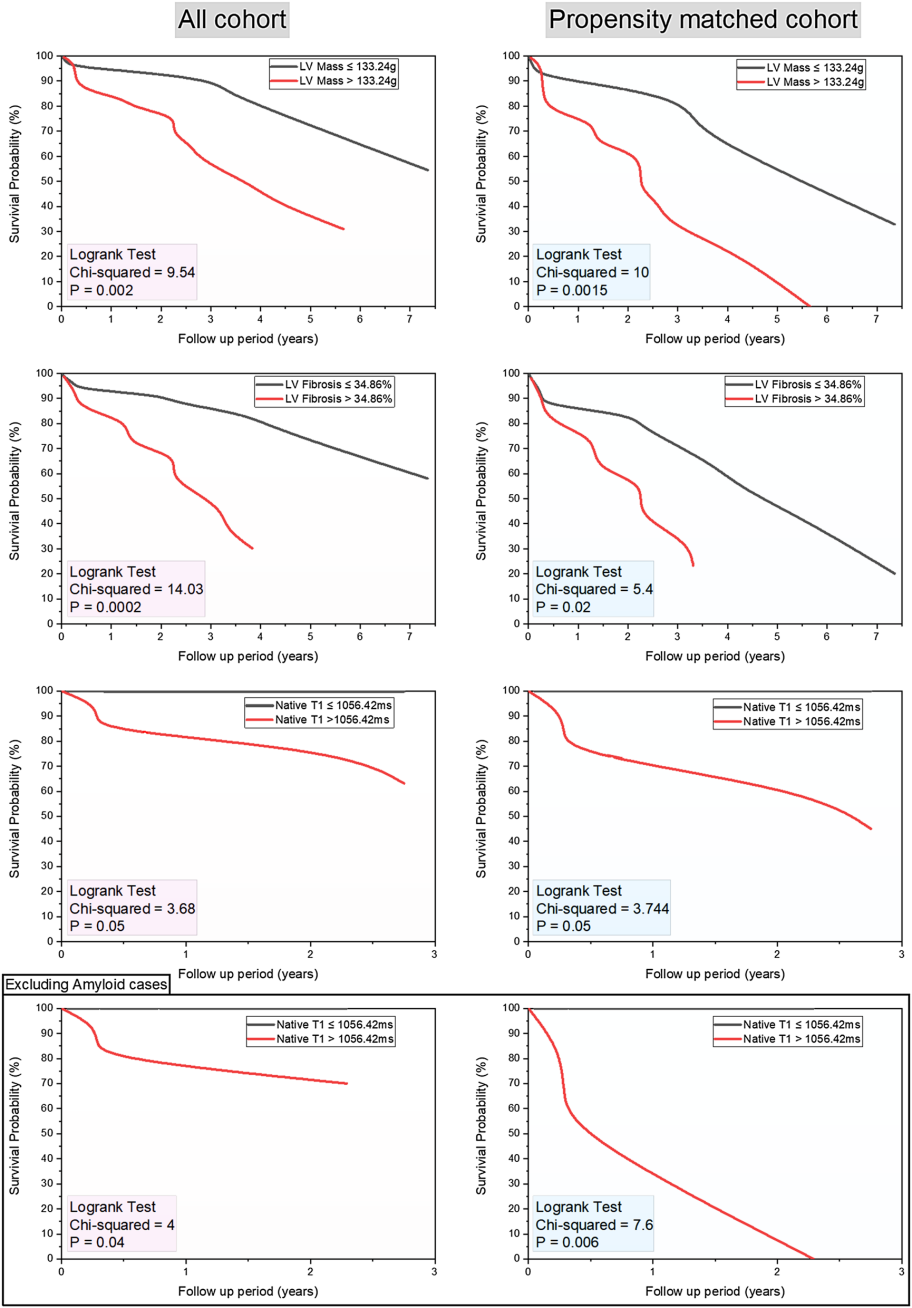
Kaplan–Meier survival analysis for variables associated with all-cause mortality. All cohort data is on the left-sided panel and the propensity matched cohort on the right-sided panel. In non-amyloid cohort, it was only native T1, which demonstrated prognostic significance.

**Figure 4:**
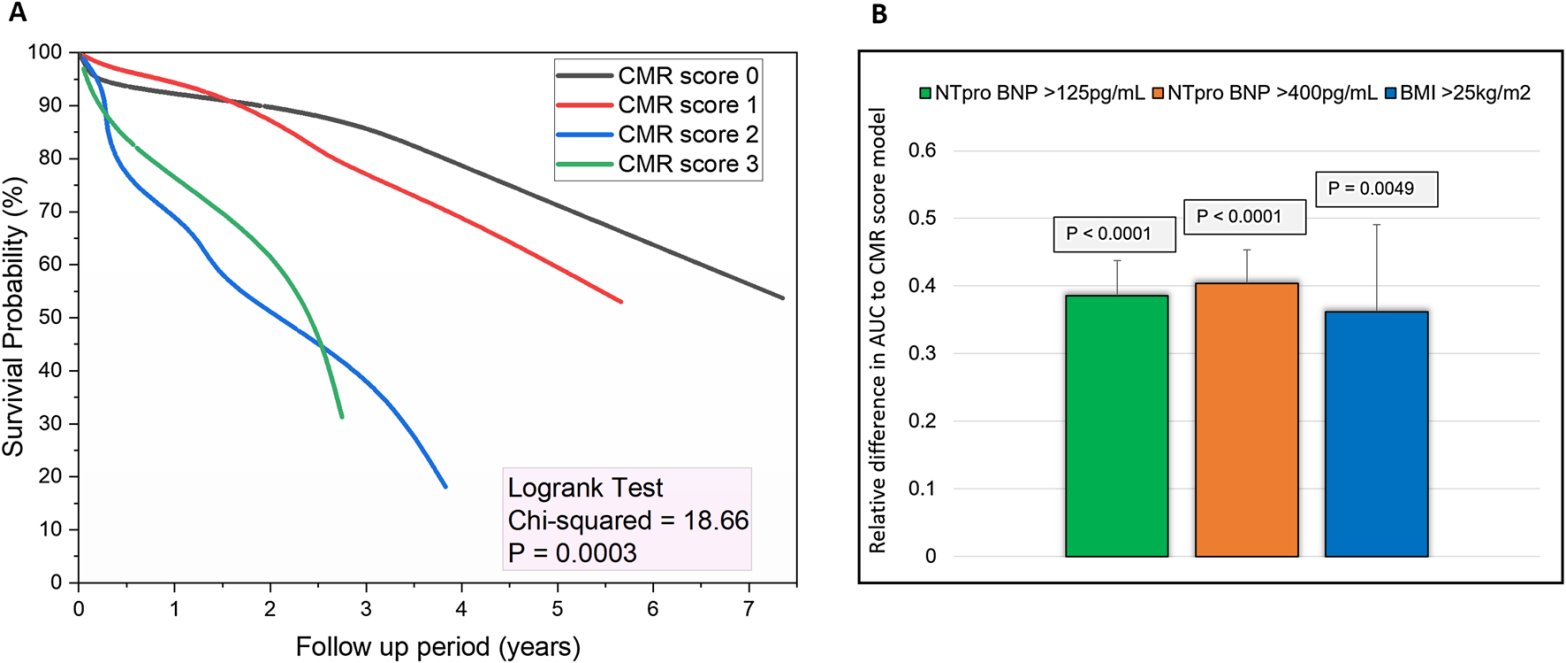
Multiparametric CMR score in HFpEF. (**A**) Kaplan–Meier survival curves. (**B**) The relative difference of area under the curve (AUC) between the CMR model and the clinical characteristics. The CMR model demonstrated significant increase in area under the curve.

For survival analysis, the mean follow-up (FU) period was 3.2 ± 2.4 years. On Kaplan Meier curves of survival probability in patients with LV mass > 133.24 g (chi-square = 9.54, p < 0.01), myocardial focal fibrosis > 34.86% (chi-square = 14.03, p < 0.01) and native T1 > 1056.42 ms (chi-square = 3.68, p = 0.05) demonstrated significantly higher all-cause mortality (Fig. 3). In addition, propensity matched analysis (n = 26) using the covariates associated with mortality (BMI and NTproBNP) still demonstrated similar results. However, in non-amyloid HFpEF cases (matched n = 19), only T1 > 1056.42 ms demonstrated higher mortality (AUC 0.833, p < 0.01). The multiparametric CMR score model demonstrated the highest weighted chi-square value in Kaplan Meier plot (chi-square = 18.66, p < 0.001) (Fig. 4A). The relative difference in AUC to predict all-cause mortality was also significantly different between the CMR score model and the clinical variables (Fig. 4B). In a multivariable cox regression comparing the CMR score model and clinical variables, CMR score model comprising these three variables independently predicted mortality in HFpEF (Table 6). In addition, the performance of the CMR score remained even when amyloid cases were excluded (Table 6).

**Table 6:** Stepwise multivariate regression in all cohort and the non-amyloid cohort.

Covariate	beta	SE	Wald	Constant	p	HR	95% CI of HR
CMR score	1.9	0.8	5.5	− 5.4	0.02	4	1.2–13.9
Variables removed in stepwise regression—BMI, NTproBNP							

				Non-amyloid HFpEF cases			
CMR score	1.96	0.9	4.9	− 5.4	0.03	3.7	1.09–12.6
Variables removed in stepwise regression—BMI							

For the whole HFpEF cohort, variables included for analysis were CMR score, BMI and NTproBNP. For the non-amyloid HFpEF cases, variables included for analysis were CMR score and BMI only.

## Discussion

In this observational study, we demonstrate that left ventricular mass assessment by CMR cine, fibrosis/scar assessment by LGE and diffuse fibrosis assessment by native T1-value are independently associated with all-cause mortality in patients with heart failure with preserved ejection fraction (HFpEF).

HFpEF is a heterogeneous disease with high morbidity and mortality ranging from 10 to 30% annually. The main risk factors for the development of HFpEF include old age, female gender, systemic hypertension, obesity, diabetes mellitus, coronary artery disease, atrial fibrillation, chronic kidney disease and chronic obstructive pulmonary disease^21^. The pathophysiology of HFpEF remains uncertain, and it may be that HFpEF is a collection of several different conditions sharing a common clinical syndrome of heart failure with preserved ejection fraction. This uncertainty may explain the absence of effective treatment. Paulus had proposed that while heart failure with reduced ejection fraction (HFrEF) is a disease of the heart with systemic manifestation, HFpEF may be a series of extracardiac vascular problems that have cardiac manifestations^22^. Thus, it is critically important to attempt to investigate and classify patients with HFpEF, in an attempt to identify high-risk patients for whom monitoring is important and for whom specific treatments may be developed.

Previous studies evaluating left ventricular hypertrophy in HFpEF observed inconsistent results. In a study by Heinzel et al*.*^6^, they demonstrated that LVH is more concentric in HFpEF patients. Furthermore, LVH can either be the cause of HFpEF as a result of multiple cardiovascular risk factors or a consequence of it. LVH contributes to vascular dysfunction and increased myocardial stiffness. LVH can be associated with fibrosis. Indeed, changes in the composition of the extracellular matrix, including increased fibrosis which in our study was associated with all-cause mortality.

Focal fibrosis quantification by LGE was associated with all-cause mortality in our cohort of patients with HFpEF. These findings are consistent with those of Kato et al*.*^23^, Murtagh et al*.*^24^ and Pöyhönen et al*.*^25^ The first study found LGE in 40 patients (36%) of 111 patients recruited. During follow up, 10 of the 111 patients (9%) experienced major cardiovascular events, including death. They defined myocardial enhancement by LGE as the signal intensity of > 2 SD above the mean signal intensity of remote myocardium. Interestingly, 8 of these 10 patients were in the LGE + group. Assessing the predictors of mortality by multivariate cox proportional hazard analysis, they found that percentage of myocardial fibrosis/scar by LGE imaging independently predicted future events with a high HR. They demonstrated that 6% burden of LV enhancement had a sensitivity of 80% and specificity of 77% to predict events. The Kaplan–Meier curves stratified by the presence of LGE and its size demonstrated its significant impact on the prognosis of patients with HFpEF (p = 0.016 by Log-rank test). The percentage of enhancement which shows association to outcomes was much higher in our study than demonstrated by Kato et al. One explanation for this is that we subgrouped our LGE enhancement assessment into fibrosis (> 2SD) and scar (> 6SD). Hence, in our study, fibrosis was only limited to > 2SD and less than 6SD. Anything above 6SD was labelled as scar.

In our study, the overall level of myocardial focal fibrosis burden by LGE that predicted all-cause mortality at a mean follow up of 3.2 ± 2.4 years was > 34.86%. These results reflect those of Murtagh et al*.*, who used CMR derived LGE in the cardiac risk stratification of patients with extracardiac sarcoidosis. LGE was present in 41 patients (20%). Amongst the 205 patients in the cohort, 12 patients (6%) died or had ventricular tachycardia (VT), 10 of these 12 patients (83%) were in the LGE + group. They estimated the death/VT rate per year was 20% higher in the LGE + group than the LGE − group (4.9% vs 0.2%, p < 0.01). However, in their work, they used the threshold of > 5SD, which is more consistent with myocardial scar quantification than fibrosis. The percentage of scar quantified by LGE that predicted the risk of death/VT events and for recognising patients with myocardial damage despite having a preserved ejection fraction was 5.7%. The plausible explanation for these differences is that they had a different patient cohort than ours. They mainly described the prognostic value of LGE imaging in cardiac sarcoidosis.

This finding was also reported by Pöyhönen et al., who evaluated the value of LGE imaging in patients suspected with non-ischaemic cardiomyopathy (NICM). In this study, the event rate for MACE was 26% in patients with LGE + versus 4% in patients without LGE (p = 0.041). Of the 86 patients involved, 15 reached the endpoint (17%), with an event rate of 7.6%/year. The highest event rate was observed in patients with LGE volume of ≥ 17%.

Thus, the presence of LGE, while not essential in the CMR diagnosis of HFpEF, defines the patients with a higher risk of major cardiovascular events, including death.

While LGE can only identify focal fibrosis, CMR T1 mapping can uncover and quantify both focal and diffuse fibrosis in the myocardium. Both native and post-contrast T1 mapping techniques have proved reliability in the diagnosis of cardiomyopathies; in predicting their prognosis, and in directing their further treatment. In our observational study, we found that native T1-value of > 1056.42 ms can independently predict all-cause mortality in patients with HFpEF. Previous studies have also investigated the role of relaxometry techniques for characterisation of myocardial tissue in HFpEF. In a study comparing native T1 values in HFpEF and controls, Kanagala et al. demonstrated that patients with HFpEF have significantly higher native T1 values (p = 0.021)^26^. However, the authors of the study did not report any association of native T1 to clinical outcomes. The plausible explanation for this was that in their study clinical outcomes mainly included HF hospitalisation versus all-cause mortality. Contrary to our study, Duca et al. also showed that native T1 times were not associated with adverse outcome (HR 1.005, 95% CI 0.99–1.01, p = 0.103) in study of 117 patients with HFpEF^27^. Moreover, in our study we also noted that T1 mapping was the only CMR parameter associated with mortality in HFpEF cohort without the diagnosis of cardiac amyloidosis. The main difference in their study was that their follow-up period was shorter at 24 months. In addition, they looked at cardiac events versus all-cause mortality in our study. Furthermore, a more direct measurement of myocardial fibrosis by quantification of extracellular volume has also demonstrated promise to further risk stratify patients with HFpEF^28,29^.

Contrary to the study by Aschauer et al*.*^30^, right ventricular systolic dysfunction (RVSD) in our cohort was not associated with an increased risk of all-cause mortality. A possible explanation for this might be that the patients we recruited are in the early stage of HFpEF in which their right heart function is not impaired.

It is worth noting that our study demonstrates the importance of further sub-phenotyping of patients with HFpEF by imaging methods. In the whole cohort, LV mass and fibrosis burden have a combined role with native T1 in predicting mortality. However, in non-amyloidosis cases, the prognostic power of LV mass and LV fibrosis was lost. This probably is associated with higher death rate in amyloid cases. Future larger but more specifically non-amyloid HFpEF studies are warranted to further evaluate the emerging role of multi-parametric CMR.

### Clinical implications

The heterogeneity of HFpEF syndrome demands for better characterisation of various HFpEF phenotypes on the basis of clinical presentation, biological and imaging data to design effective therapies^31^. In our study, we identified LVH by multivariable CMR, scar/fibrosis by LGE and diffuse fibrosis by native T1-mapping as three CMR derived markers for all-cause mortality in HFpEF.

Therefore, CMR is not only capable of probing the possible aetiology and pathogenesis of HFpEF; but it is capable of providing us with markers of increased risk of mortality, thus enabling us to risk-stratify patients with HFpEF in whom new therapeutic interventions could be directed. We do call for a reconsideration of the diagnostic algorithm of patients with HFpEF to routinely include CMR to assist therefore in defining the aetiology and identifying the patients with a higher risk of mortality; on whom further therapeutic interventions should concentrate.

We do know the association between myocardial fibrosis and at least two of the three markers we identified as predictors of risk of mortality in HFpEF. Besides, it is well known that in patients with heart failure with reduced ejection fraction, myocardial fibrosis predicts the incidence of cardiac arrhythmias.

We propose that our results should be confirmed prospectively along with an assessment of the arrhythmia burden in these patients with HFpEF and evidence of fibrosis or LVH. Once both, the impact of fibrosis and LVH on arrhythmia burden in HFpEF and on patients' mortality is confirmed, we do believe that therapeutic interventions such as beta-blockers and device therapy could be tested in patients with HFpEF in a randomised controlled trial. Such a proposed strategy could avoid the previous pitfalls that adversely affected many trials of therapy for HFpEF.

### Limitations

There are several limitations to our study. Firstly, this is an observational study from a single centre. Future prospectively designed studies need to confirm our findings. Nevertheless, the patients recruited in this study are from the real clinical world, and the results overall imply a clear advantage of using CMR for prognostication in HFpEF. Another limitation of this study is we only recruited patients who had CMR and the request for CMR was at clinical discretion. This has the potential to introduce selection bias in this study. This study did not record therapeutic interventions, which may provide further insight into prognosis. This study used optimum cut-offs for the CMR variables which may be centre specific, and caution should be applied in using these. Future larger HFpEF studies are warranted to derive more generic cut-offs. Finally, in this study, we also excluded patients who have unstable symptoms and are not able to lie flat because of shortness of breath. These patients are more likely to represent a higher risk group with more adverse prognosis.

## Conclusion

In patients with HFpEF, multiparametric CMR aids prognostication. Our results show that left ventricular fibrosis and hypertrophy quantified by CMR are associated with all-cause mortality in patients with HFpEF.

## Data Availability

The datasets used and/or analysed during the current study are available from the corresponding author on reasonable request.

## Abbreviations

AUC: Area under the curve
CAD: Coronary artery disease
CKD: Chronic kidney disease
COPD: Chronic obstructive pulmonary disease
CMR: Cardiac magnetic resonance
DM: Diabetes mellitus
eGFR: Estimated glomerular filtration rate
EF: Ejection fraction
HF: Heart failure
HFpEF: Heart failure with preserved ejection fraction
HFrEF: Heart failure with reduced ejection fraction
LGE: Late gadolinium enhancement
LVEDV: Left ventricular end-diastolic volume
LVEF: Left ventricular ejection fraction
LVESV: Left ventricular end-systolic volume
LVH: Left ventricular hypertrophy
MACE: Major adverse cardiovascular events
MI: Myocardial infarction
MOLLI: Modified look-locker inversion recovery
NT-proBNP: N-Terminal pro-brain-type natriuretic peptide
ROC: Receiver operating characteristic curve
RVEDV: Right ventricular end-diastolic volume
RVESV: Right ventricular end-systolic volume
RVSD: Right ventricular systolic dysfunction
SSFP: Steady-state free precession
